# Enriching the environment to disinhibit the brain and improve cognition

**DOI:** 10.3389/fncel.2012.00053

**Published:** 2012-11-15

**Authors:** Arianna Maffei

**Affiliations:** Department of Neurobiology and Behavior, SUNY-Stony BrookStony Brook, NY, USA

**A commentary on**

**Environmental enrichment decreases GABAergic inhibition and improves cognitive abilities, synaptic plasticity, and visual functions in a mouse model of Down syndrome**

by Begenisic, T., Spolidoro, M., Braschi, C., Baroncelli, L., Milanese, M., Pietra, G., Fabbri, M. E., Bonanno, G., Cioni, G., Maffei, L., and Sale, A. (2011). Front. Cell. Neurosci. 5:29. doi: 10.3389/fncel.2011.00029

Rearing animals in stimulating environments affects the morphology (Diamond et al., 1966), chemistry (Bennett et al., 1964), and physiology (Kiyono et al., 1981) of their brain. This experimental manipulation, Environmental Enrichment (EE), is used to investigate influences of increased sensory-motor stimulation on brain and behavior (Curtis and Nelson, 2003).

Studies using EE identified molecular, anatomical, physiological, and functional events occurring as a consequence of enriched interactions of an experimental subject with its environment. The effects of EE on brain function are highly impactful and led to critical reassessments of the importance of cognitive stimulation early in life to improve academic achievement (Curtis and Nelson, 2003) and of the need for cognitive stimulation for preventing neurodegenerative disorders (Greenwood, 2007).

EE increases sensory-motor stimulation in a non-invasive, non-pharmacological fashion, rendering it particularly interesting from a clinical perspective. Animal models of neurodegenerative and neurodevelopmental disorders showed marked improvement in memory tasks (van Praag et al., 2000; Bennett et al., 2006; Kumar et al., 2012; Redolat and Mesa-Gresa, 2012). These effects were often associated with changes in the morphological complexity of pyramidal neurons and with increased neurogenesis in adult subjects when EE was paired with physical exercise (Pang and Hannan, 2013). Thus, EE may promote recovery of cognitive functions by extensively rewiring neural circuits (van Praag et al., 2000).

Begenisic and collaborators applied EE to a well established mouse model (the Ts65D) of Down syndrome (Begenisic et al., 2011), a condition due to trisomy of chromosome 21 in humans. The study of EE in this context is highly relevant to public health, as Down syndrome is the most common genetic cause of mental retardation (Roizen and Patterson, 2003). Initial studies of the mechanisms underlying mental retardation in animal models of Down syndrome focused on the effects of the mutations on hippocampal long term synaptic plasticity (Siarey et al., 1997), a cellular correlate of learning and memory. Recent work using the Ts65D model demonstrated that GABA signaling is *also* increased (Best et al., 2007; Belichenko et al., 2009). A shift in the balance between glutamatergic and GABAergic signaling to favor the latter may thus contribute significantly to the cognitive symptoms reported. In favor of this hypothesis, pharmacological reduction of GABA-mediated inhibition in the Ts65D mouse model reversed deficits in hippocampus-mediated memory tasks (Fernandez et al., 2007).

*In rodents*, one of the effects of EE on brain circuits is to reduce inhibitory synaptic transmission mediated by GABA receptors (Sale et al., 2007). *Thus, EE could in principle be used to restore healthy levels of neural activity, in the Ts65D mouse and possibly in other disorders characterized by increased inhibition* (Wong et al., 2006; Hines et al., 2008; Sun et al., 2009). Begenisic and collaborators tested this possibility in the Ts65D model of Down syndrome and found that EE does indeed promote the recovery of spatial memory abilities as well as the capability for plasticity in the hippocampus (Begenisic et al., 2011). Besides cognitive deficits, the Ts65D model shows significant impairments in visual acuity and ocular dominance (Begenisic et al., 2011). Defects in visual function are typical of Down syndrome pathophysiology (John et al., 2004), thus the Ts65D model recapitulates not only the cognitive but also the sensory deficits of the disorder and may offer the unique opportunity to test whether the same manipulation can restore both functions. Changes in inhibition are associated with decreased visual acuity and altered ocular dominance in response to visual deprivation (Hensch et al., 1998; Hensch, 2005). EE restores both these visual functions by reducing inhibition in visually deprived rodents (Sale et al., 2007). Interestingly, Begenisic et al show that the hippocampus and visual cortex of Ts65D mice have similar defects in the evoked release of GABA (Begenisic et al., 2011). Thus, alterations of GABA levels are likely to have a broad impact on both cognitive and sensory function and may be affected similarly by EE. In an elegant set of experiments Begenisic and collaborators show that EE restores hippocampus based memory tasks, visual function and levels of GABA release from synaptosomes to control levels (Begenisic et al., 2011). In visual cortex EE and reduced GABA transmission are restore *the capacity for* plasticity in the adult brain (Tognini et al., 2012), while in the hippocampus these processes are associated with recovery of successful performance in memory tasks and of long term plasticity at glutamatergic synapses (Eckert and Abraham, 2012).

The results in Begenisic et al. are highly relevant to the biomedical field as they indicate that a non-invasive practice can improve sensory and cognitive deficits associated with changes in inhibitory transmission. This effect is not limited to a specific developmental window but can occur in the adult brain. EE offers a possible alternative approach to the pharmacological reduction of inhibition in patients Down syndrome. These patients are more prone to convulsions than the general population (Menendez, 2005), and antagonists of the GABA system are known pro-convulsive drugs (Ben-Ari, 2006). While effective and safer compounds may be developed in the near future, the effect of EE on GABA signaling offers the great advantage of using behavioral modifications to modulate the balance between excitation and inhibition (Sale et al., 2007). This approach has the potential to help rescue neural circuit function, while limiting possible loss of network stability (Koh et al., 2007) that may occur as consequence of global modulation of neurotransmitter systems (Nabbout, 2001). The effects of EE are not limited to a reduction in GABA signaling, as changes in morphology and physiology of neurons are also reported (Nithianantharajah and Hannan, 2006). Thus, intense sensory-motor stimulation may offer therapeutic aid for a number of neurological disorders in addition to those characterized by increased inhibition (Nithianantharajah and Hannan, 2006; Pang and Hannan, 2013).

To play devil's advocate, one may argue that animal models are usually reared in conditions that are not highly stimulating, while humans with or without Down syndrome are exposed to complex sensory and cognitive stimuli in their daily life. Thus, EE may not provide as big an improvement on cognitive deficits as suggested by animal research. However, a large body of work shows that increased sensory and cognitive stimulation in early life support increased learning in healthy school children (Curtis and Nelson, 2003) and continued brain stimulation throughout life through exposure to novel *stimuli and* learning experiences decreases the incidence of memory loss and dementia in aging individuals (Fratiglioni et al., 2004). Thus, the impact of EE on public health may be broader than expected, benefiting not only the population of patients with mental retardation, but also to the general public by promoting healthy brain development and preventing *aging-related cognitive deficits* (Nithianantharajah and Hannan, 2006; Pang and Hannan, 2013).
